# Circulating microRNAs as biomarkers for Sepsis secondary to pneumonia diagnosed via Sepsis 3.0

**DOI:** 10.1186/s12890-019-0836-4

**Published:** 2019-05-14

**Authors:** Wenping Zhang, Jianchao Jia, Zi Liu, Dan Si, Lijun Ma, Guojun Zhang

**Affiliations:** 1 Department of Respiratory and Critical Care Medicine, The People’s Hospital of Zhengzhou University, Zhengzhou, Henan China; 2 Department of Respiratory and Critical Care Medicine, The First Affiliated Hospital of Zhengzhou University, No.1, East Jianshe Road, Zhengzhou, 450052 Henan China

**Keywords:** Sepsis, SOFA score, Pneumonia, miR-7110-5p, miR-223-3p, Sepsis 3.0

## Abstract

**Background:**

Sepsis biomarkers have limited specificity and sensitivity. Few studies have investigated microRNA (miRNA) biomarkers for sepsis secondary to pneumonia. This study aims to investigate the diagnostic and prognostic values of miRNAs in sepsis secondary to pneumonia.

**Methods:**

Sepsis 3.0 was used to diagnose sepsis. Screening was performed through the Agilent miRNA chip technology by using the following criteria: *p* < 0.05, fold ≥2 or < 0.5, or copy number > 50 change. This study recruited 52 patients with pneumonia, including 31 males (59.6%) and 21 females (40.4%), 44 patients with sepsis secondary to pneumonia were diagnosed via Sepsis 3.0 (34 [77.3%] males and 10 [22.7%] females), and 21 healthy controls were used for miRNA verification. The miRNA levels were detected through fluorescence real-time quantitative polymerase chain reaction (qRT-PCR). Results: Fluorescence qRT-PCR detection showed that the miR-7110-5p and miR-223-3p expression levels in both patient groups were upregulated compared with those in the healthy controls. The expression levels differed between patients with pneumonia and those with sepsis secondary to pneumonia. The sensitivity and specificity of miR-7110-5p to differentiate sepsis from healthy controls were 84.2 and 90.5%, whereas those of miR-223-3p were 82.9 and 100%, respectively. Multivariate analysis of variance suggested that the presence of sepsis affected the miR-223-3p level (*p* = 0.041), whereas the presence of sepsis (*p* = 0.000) and the underlying disease (*p* = 0.025) influenced the miR-7110-5p level.

**Conclusions:**

MiR-223-3p could be utilized to predict sepsis secondary to pneumonia.

## Background

Sepsis is a common complication that involves severe trauma, shock, infection, and surgery; it can lead to septic shock and multiple organ dysfunction syndrome (MODS). Sepsis is also a major cause of death in critical patients. It remains a remarkable challenge in clinical medicine because of its high mortality and morbidity rates. In developed countries, the incidence of sepsis reaches 100/100,000 [1], and approximately 2% of hospitalized patients are diagnosed during admission [2, 3]. With advancements in critical medicine, evidence-based medicine is widely used in clinical practices to address sepsis and its related conditions. However, approximately 1/5–1/2 of the patient population with sepsis dies of MODS [4, 5].

MicroRNAs (miRNAs) are endogenous noncoding small RNAs of approximately 21–25 nucleotides in length. In 1993, Lee [6] discovered the first miRNA (lin-4) miRNAs in new *Caenorhabditis* species. Since then, thousands of miRNAs have been found. miRNAs can inhibit post-transcriptional gene expression or promote targeted mRNA degradation but cannot encode a protein. miRNAs can be detected in different fluids, such as blood, sweat, and urine. Whole panels of deregulated miRNAs have been described in the blood of patients with inflammatory/infectious diseases, thereby suggesting that circulating miRNAs might be suitable biomarkers in sepsis [7] but not in patients with respiratory infection. This study aimed to identify the differences in microRNA expression in patients affected with pneumonia and with sepsis secondary to pneumonia.

## Methods

### Subject

We conducted a prospective observational study to identify the differences in microRNA expression in patients affected with pneumonia and with sepsis secondary to pneumonia. An Agilent miRNA chip was used to screen the plasma of five patients with pneumonia and five patients with sepsis secondary to pneumonia. Fluorescence quantitative reverse-transcription polymerase chain reaction (qRT-PCR) was applied to test the differences in miRNA levels among the patients with pneumonia, those with sepsis secondary to pneumonia, and the control group, which were included from August 2016 to January 2017. Pneumonia was diagnosed in accordance with the diagnosis and treatment guidelines of hospital-acquired pneumonia (HAP) and community-acquired pneumonia (CAP) drafted by the American Thoracic Society and the Infectious Diseases Society of America in 2007 and 2016 [8, 9]. Sepsis secondary to pneumonia was diagnosed based on the Sepsis 3.0 guideline, which can be simplified as sepsis = infection + sequential organ failure assessment (SOFA) ≥ 2 [10]. We used an adjudication procedure according to the guideline. For each case, three respiratory specialists independently conducted the diagnosis, and the cases with consistent diagnosis were included in the analysis. The Ethics Committee approved this study. All patients or guardians signed an informed consent form. Patients were excluded according to the following criteria: 1) age < 18 years; 2) death within 24 h of admission; 3) neutrophil count ≤0.5 × 10^9^/L; 4) HIV/AIDS; and 5) unwillingness to participate in this study.

### miRNA gene chip analysis

Agilent Human miRNA chip V21.0 provided data from the miRNA database (miRBase) version 21.0 and covered 2661 human-related miRNAs. We conducted statistical analysis control to avoid false discovery caused by individual bias and experimental factors. The coefficient of variance (CV) for the repeated probe (10 repetitions) signal was not more than 7% with an acceptance value of < 15%. Horizontal evaluation of the chip quality suggested that the chip was stable. Cluster analysis and biology repeat sample correlation analysis showed that the samples in the pneumonia group and the other group exhibited good correlation.

qRT-PCR analysis was conducted to detect the expression levels of the selected miRNAs in healthy controls, patients with pneumonia, and patients with sepsis secondary to pneumonia. RNA was extracted from the plasma by using an miRcute serum/plasma miRNA isolation kit (TianGen, Biotech Company, Beijing, China), which relatively enriched the small RNAs. qRT-PCR analysis was also performed using SYBR Green I fluorescent dye in a miScript SYBR® Green PCR kit (Qiagen, Valencia, CA). All procedures were conducted in accordance with the manufacturer’s instructions and were repeated thrice. The reverse primer in the miRcute miRNA qPCR detection kit (SYBR) was similar for all miRNAs, but the forward primers used were different. The forward primers for the miRNAs were synthesized by the Sangon Biotech Company. An ABI PRISM 7300 detection system was utilized for qRT-PCR analysis. The miRNA sequence is listed in Table 1.

**Table 1:** miRNAs and Primer sequence

miRNAs	miRNAs sequences	Primer
hsa-miR-4800-5p	AGUGGACCGAGGAAGGAAGGA	AGTGGACCGAGGAAGGAAGGA
hsa-miR-6510-5p	CAGCAGGGGAGAGAGAGGAGUC	CAGCAGGGGAGAGAGAGGAGTC
hsa-miR-6740-5p	AGUUUGGGAUGGAGAGAGGAGA	AGTTTGGGATGGAGAGAGGAGA
hsa-miR-7110-5p	UGGGGGUGUGGGGAGAGAGAG	TGGGGGTGTGGGGAGAGAGAG
hsa-miR-765	UGGAGGAGAAGGAAGGUGAUG	TGGAGGAGAAGGAAGGTGATG
hsa-miR-940	AAGGCAGGGCCCCCGCUCCCC	AAGGCAGGGCCCCCGCTCCCC
hsa-miR-150-5p	UCUCCCAACCCUUGUACCAGUG	TCTCCCAACCCTTGTACCAGTG
hsa-miR-223-3p	UGUCAGUUUGUCAAAUACCCCA	UGUCAGUUUGUCAAAUACCCCA
hsa-miR-122-5p	UGGAGUGUGACAAUGGUGUUUG	TGGAGTGTGACAATGGTGTTTG
hsa-miR-25-3p	CAUUGCACUUGUCUCGGUCUGA	CATTGCACTTGTCTCGGTCTGA
hsa-miR-16-5p	UAGCAGCACGUAAAUAUUGGCG	TAGCAGCACGTAAATATTGGCG
miR universal primer R		GATCCAGTCTCAGGGTCCGAG

### Statistical analysis

Data were statistically analyzed using SPSS 22.0. In the univariate analysis, data with normal and non-normal distributions and the count data were evaluated through t-test, Mann–Whitney test, and χ^2^ test, respectively. *p* < 0.05 indicated significant differences. The specificity and sensitivity of miRNAs in the diagnosis of sepsis were calculated in terms of the area under the receiver operating characteristic (ROC) curve.

## Results

### miRNA gene chip analysis

**Fig. 1:**
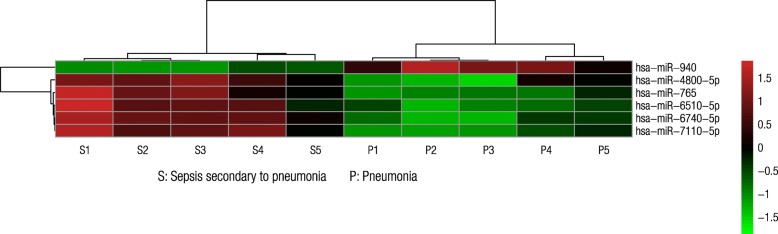
Heatmap of the differential expression of miRNAs (*p* < 0.01). The expression levels of miR-4800-5p, miR-6510-5p, miR-6740-5p, miR-7110-5p, and miR-765 were upregulated in the group with sepsis secondary to pneumonia. The miR-940 expression was downregulated in the sepsis group

Six miRNAs, namely, miR-4800-5p, miR-6510-5p, miR-6740-5p, miR-7110-5p, miR-765, and miR-940, satisfied the screening criteria of *p* < 0.01, fold change ≥2 or < 0.5, and copy number > 50. Among these miRNAs, miR-940 expression was downregulated in the sepsis group (Fig. 1). Two miRNAs, namely, miR-6740-5p and miR-7110-5p, also met the screening criteria and were upregulated in the sepsis group.

### Clinical characteristics of the subjects

Exactly 52 patients with pneumonia, including 31 males (59.6%) and 21 females (40.4%) with an average age of 52.10 ± 16.15 years old, were enrolled in this study. Fifty (96.1%) patients were diagnosed with CAP, and two patients (3.8%) were diagnosed with HAP. Exactly 44 patients with sepsis secondary to pneumonia, including 34 males (77.3%) and 10 females (22.7%) with an average age of 63.72 ± 19.83 years old, were diagnosed by applying the Sepsis 3.0 criteria. Exactly 29 patients (65.9%) were diagnosed with CAP, and 15 patients (34.1%) were diagnosed with HAP/VAP (ventilator-associated pneumonia). Among the 44 sepsis cases, 42 (95.5%) had a definite etiology. They were due to bacteria (23, 52.3%), viruses (4, 9.1%), *Aspergillus* (2, 4.5%), and multiple pathogens (13, 29.5%). Among the sepsis cases with multiple pathogens, seven cases were attributed to bacteria with fungi. Three cases were attributed to more than two different bacterial strains. Two cases were attributed to virus with fungi, and one case to bacteria with virus. The SOFA score of patients with sepsis secondary to pneumonia was 6.47 ± 3.11. The clinical characteristics of all patients are presented in Table 2. Exactly 21 healthy controls were volunteer doctors and nurses in our hospital.

**Table 2:** Clinical characteristic of patients with pneumonia and sepsis secondary to pneumonia

		Pneumonia (*n*)	Sepsis secondary to pneumonia (*n*)	*P* value
Sex	Male	31	34	0.065
	Female	21	10	
Age		52.10 ± 16.15	63.72 ± 19.83	0.002
Merges more than 1 kind of underlying disease		6 (11.50%)	34 (77.30%)	0.000
SOFA score			6.47 ± 3.11	
SOFA score above baseline			5.84 ± 2.92	
Prognosis	Survivor	52 (100%)	23 (52.30%)	
	Dead	0 (0%)	21 (47.70%)	
Major underlying comorbidities	Diabetes	0	9 (20.45%)	
	Acute cerebral infarction or cerebral hemorrhage	2 (3.85%)	9 (20.45%)	
	chronic renal insufficiency	3 (3.85%)	5 (11.36%)	
	coronary heart disease	0	5 (11.36%)	
	autoimmune disease	1 (1.92%)	3 (6.82%)	
	Chest trauma or surgery	0	3 (6.82%)	

### RT-PCR validation of miRNAs

**Fig. 2:**
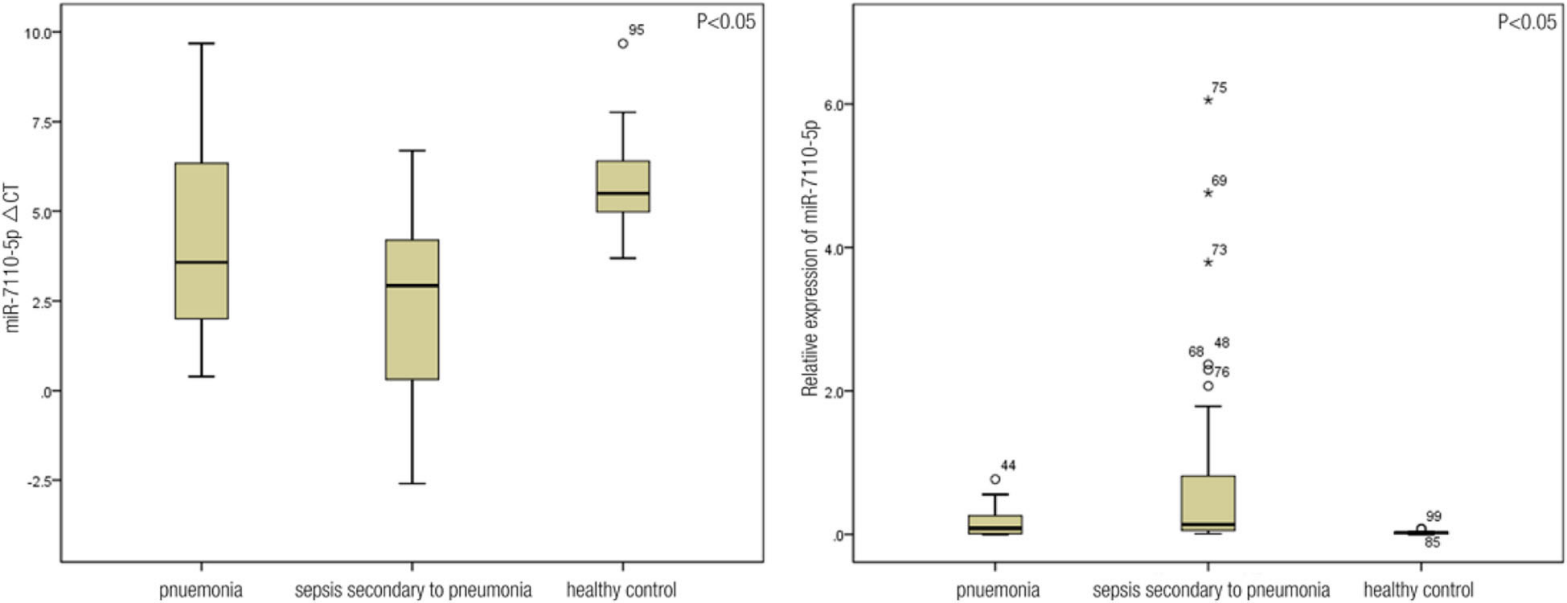
Image on the left shows that the ∆CT values of miR-7110-5p in the pneumonia, sepsis, and healthy control groups were 4.15 ± 2.52, 2.27 ± 2.64, and 5.73 ± 1.35, respectively, which were significantly different (*p* = 0.000). Moreover, the ∆CT of miR-7110-5p significantly differed between the healthy control and pneumonia groups (*p* = 0.002), healthy control and sepsis groups (*p* = 0.000), and pneumonia and sepsis groups (*p* = 0.001). Image on the right shows that the relative expression levels of miR-7110-5p in the three groups were 0.084 (0.012, 0.28), 0.133 (0.054, 0.963), and 0.022 (0.011, 0.033), which were significantly different (*p* = 0.000). Moreover, the relative expression of miR-7110-5p significantly differed between healthy control and pneumonia groups (*p* = 0.015), healthy control and sepsis groups (*p* = 0.000), and pneumonia group and sepsis groups (*p* = 0.006)

**Fig. 3:**
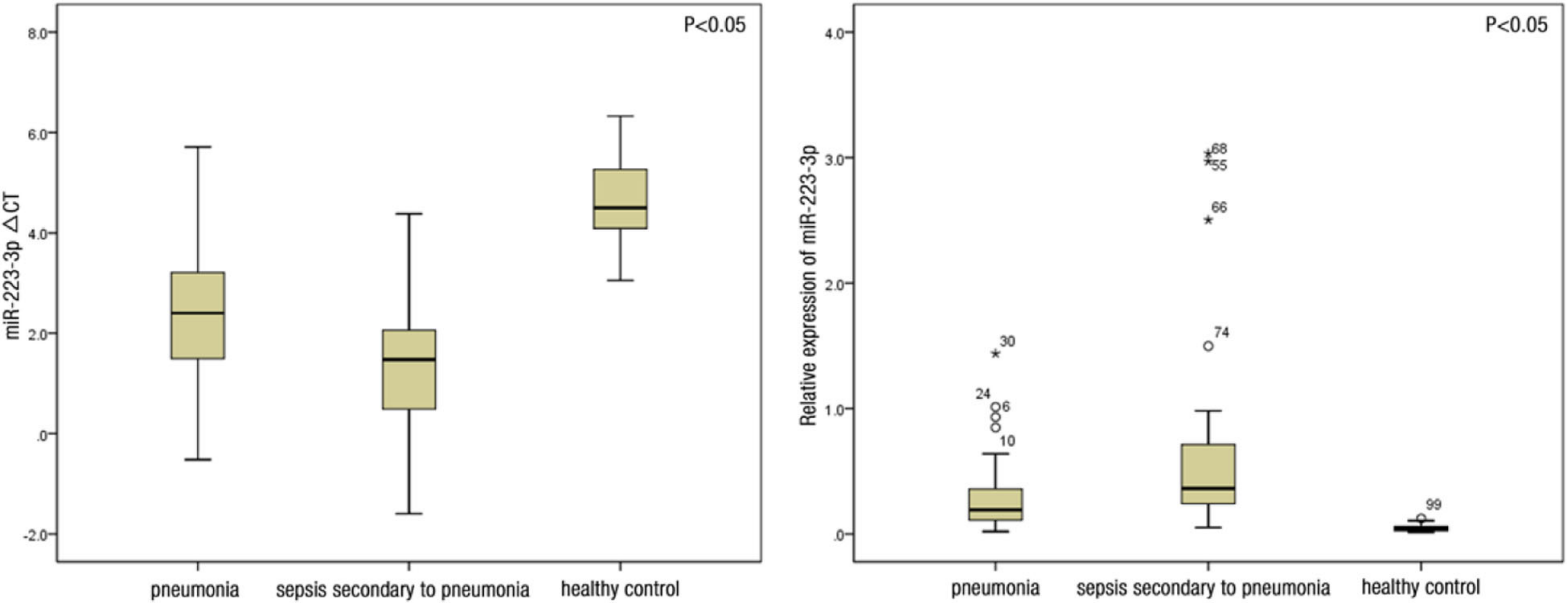
Image on the left shows that the ∆CT levels of miR-223-3p in the pneumonia, sepsis, and healthy control groups were 2.39 ± 1.36, 1.44 ± 1.43, and 4.58 ± 0.91, respectively, which were significantly different (*p* = 0.000). The ∆CT values of miR-223-3p significantly differed between the healthy and pneumonia groups (*p* = 0.000), healthy control and sepsis groups (*p* = 0.000), pneumonia and sepsis groups (*p* = 0.002). Image on the right shows that the relative expression levels of miR-223-3p in the three groups were 0.189 (0.107, 0.367), 0.361 (0.221, 0.735), and 0.044 (0.022, 0.061), which were significantly different (*p* = 0.000). In addition, the relative expression level of miR-223-3p significantly differed between the healthy control and pneumonia groups (*p* = 0.000), healthy control and sepsis groups (*p* = 0.000), and pneumonia and sepsis groups (*p* = 0.004)

The results of the gene chip analysis indicated the presence of miR-4800-5p, miR-6510-5p, miR-6740-5p, miR-7110-5p, miR-765, and miR-940, which were rarely mentioned in previously published data. In this regard, relative data regarding miRNAs in sepsis were reviewed. miR-150, miR-223-3p, miR-25, and miR-122, which have been intensively studied but remained controversial in terms of their relationship with sepsis, were selected for RT-PCR validation. Ten miRNAs, including miR-4800-5p, miR-6510-5p, miR-6740-5p, miR-7110-5p, miR-765, and miR-940 satisfied the screening criteria and were selected as primary genes for the tests. The four remaining miRNAs, namely, miR-150, miR-223-3p, miR-25, and miR-122 were related to sepsis. miR-16 was used as the reference gene. Table 3 shows the ΔCT values of the 10 miRNAs in the healthy controls, patients with pneumonia, and patients with sepsis secondary to pneumonia. qRT-PCR results indicated that the expression levels of miR-7110-5p and miR-223-3p differed between the two patient groups and were upregulated in the plasma of patients with sepsis secondary to pneumonia. miR-7110-5p and miR-223-3p expression levels were higher in patients with pneumonia and sepsis than those in healthy controls (Figs. 2 and 3).

**Table 3:** RT-PCR of the 10 miRNAs in patients with pneumonia and sepsis secondary to pneumonia

	ΔCT		*P* value
	Pneumonia	Sepsis secondary to pneumonia	
miR-223-3p	2.39 ± 1.36	1.44 ± 1.43	0.002
miR-7110-5p	4.15 ± 2.52	2.27 ± 2.64	0.001
miR-940	3.19 ± 1.84	2.92 ± 1.96	0.493
miR-4800-5p	6.08 ± 2.81	6.11 ± 2.54	0.967
miR-150	4.29 ± 2.11	4.66 ± 1.90	0.406
miR-25	−2.28 ± 1.86	−2.33 ± 2.10	0.914
miR-122	2.70 ± 2.30	2.20 ± 2.06	0.332
miR-6740-5p	5.15 ± 3.00	6.03 ± 3.15	0.231
miR-765	5.06 ± 3.12	5.24 ± 2.97	0.822
miR-6510-5p	3.62 ± 1.74	3.77 ± 1.83	0.756

Considering the differences in the age and presence of comorbidities in pneumonia versus sepsis due to pneumonia, multivariate analysis of variance (MANOVA) was conducted to affirm whether the differences in miRNA expression were due to the presence of sepsis. In MANOVA, the variable factors include age ≥ 60 years or not, merged underlying disease or not, and sepsis or not. The presence of sepsis affected the level of miR-223-3P (*p* = 0.041), whereas the presence of sepsis (*p* = 0.000) and underlying disease (*p* = 0.025) affected the level of miR-7110-5p. Further analysis cannot be conducted, because the variety of underlying diseases and number of cases are limited.

### Identification of diagnostic and prognostic predictors for sepsis

**Fig. 4:**
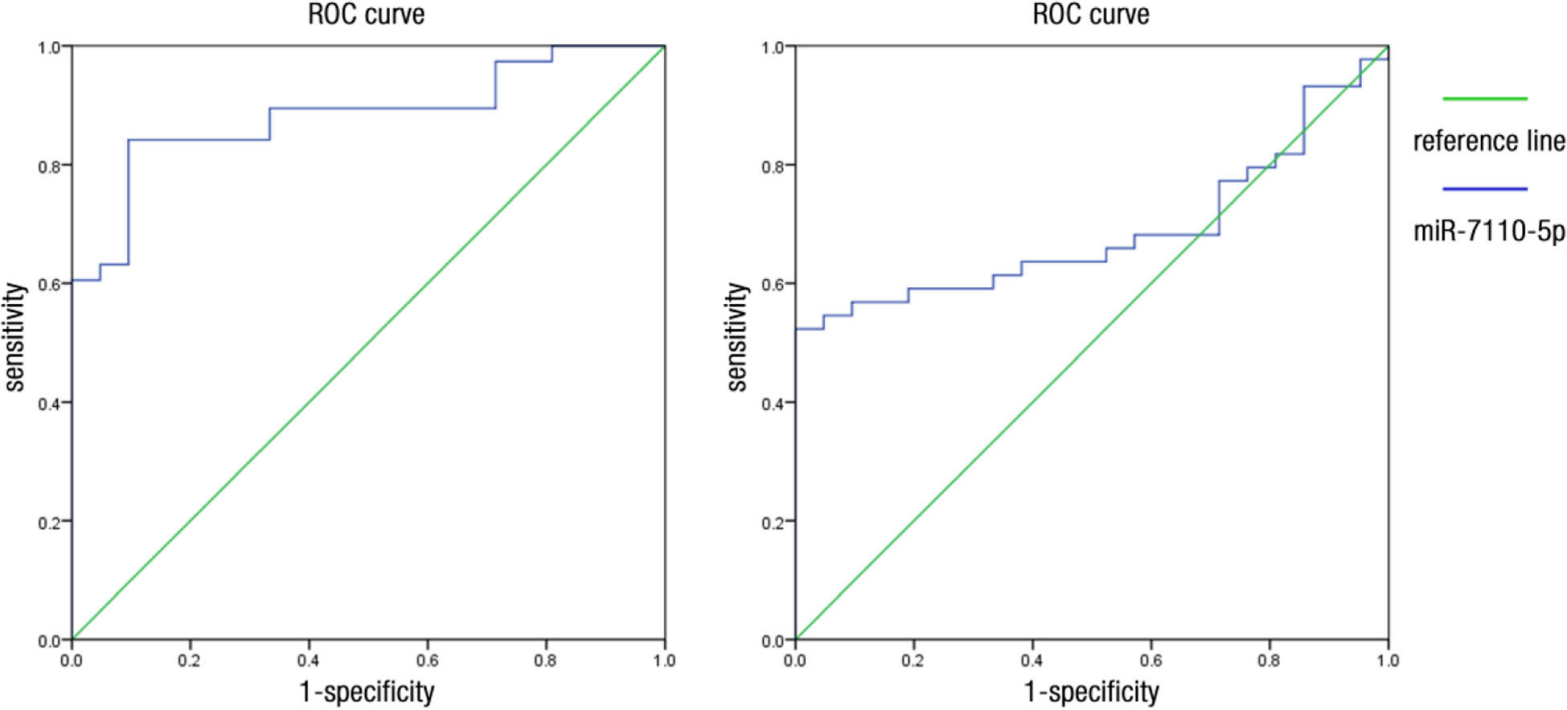
ROC curve of miR-7110-5p. Image on the left shows that the AUC of the ROC of miR-7110-5p for the prediction of pneumonia was 0.687. Image on the right shows that the AUC of the ROC of miR-7110-5p for sepsis prediction secondary to pneumonia was 0.883

**Fig. 5:**
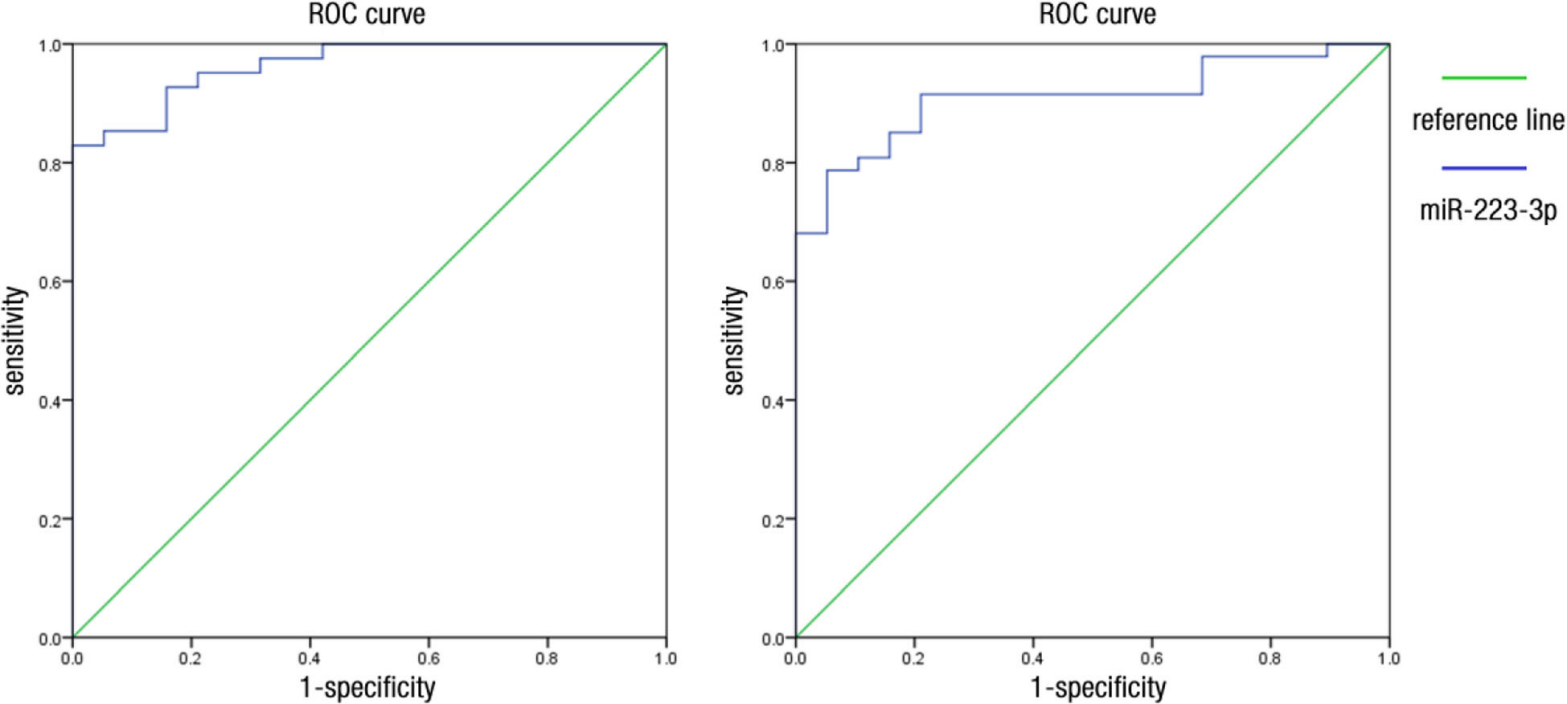
ROC curve of miR-223-3p. Image on the left shows that the AUC of the ROC of miR-223-3p for the prediction of pneumonia was 0.909. Image on the right shows that the AUC of the ROC of miR-223-3p for sepsis prediction secondary to pneumonia was 0.964

The area under the curve (AUC) of the ROC of miR-7110-5p for the prediction of pneumonia was 0.687 (Fig. 4). At a cutoff value of 3.66, miR-7110-5p yielded a sensitivity of 52.3% and a specificity of 100%. The AUC of the ROC of miR-7110-5p for the prediction of sepsis secondary to pneumonia was 0.883 (Fig. 4). At a cutoff value of 4.41, miR-7110-5p yielded a sensitivity of 84.2% and a specificity of 90.5%. The AUC of the miR-223-3p ROC for the prediction of pneumonia was 0.909 (Fig. 5). At a cutoff value of 3.246, miR-223-3p yielded a sensitivity of 78.7% and a specificity of 94.7%. The AUC of the miR-223-3p ROC for the prediction of sepsis secondary to pneumonia was 0.964 (Fig. 5). At a cutoff value of 2.759, miR-223-3p yielded a sensitivity of 82.9% and a specificity of 100%.

The SOFA score could indicate the severity of sepsis. The ΔCT of miR-223-3p was 1.763 ± 1.401 at SOFA scores ≤6 and was 0.762 ± 1.308 at SOFA scores of > 6. The scores significantly differed between the two patient groups (*p* < 0.05). The serum expression of miR-7110-5p did not significantly differ for patients with various SOFA scores.

Among the 44 enrolled patients with sepsis secondary to pneumonia, 23 survived (52.3%), whereas 21 died (47.7%). Table 4 shows the clinical characteristics of patients with sepsis. The plasma levels of miR-7110-5p and miR-223-3p did not significantly differ between the patients who survived and died of sepsis (Table 5).

**Table 4:** Clinical characteristic of 44 cases of sepsis secondary to pneumonia

		Survivor (*n*)	Dead (*n*)	*P* value
Sex	Male	21	13	
	Female	2	8	
Age		57.30 ± 21.51	70.76 ± 15.40	0.023
SOFA score		5 (4,7)	7 (4,9.5)	0.010
SOFA above baseline		5 (3,6)	7 (4,9)	0.016
SOFA score (oxygenation index)		2 (2,3)	3 (2.5,4)	0.024
coagulation disorder	No	14 (60.9%)	10 (47.6%)	0.378
	Yes	9 (39.1%)	11 (52.4%)	
liver dysfunction	No	17 (73.9%)	19 (90.5%)	0.245
	Yes	6 (26.1%)	2 (9.5%)	
circulatory dysfunction	No	18 (78.3%)	13 (61.9%)	0.325
	Yes	5 (21.7%)	8 (47.7%)	
disturbance of consciousness	No	15 (65.2%)	8 (38.1%)	0.070
	Yes	8 (34.8%)	13 (61.9%)	
renal dysfunction	No	17 (73.9%)	12 (57.1%)	0.240
	Yes	6 (26.1%)	9 (42.9%)	
PCT (ng/mL)		0.64 (0.21,1.86)	0.99 (0.51,8.70)	0.109
WBC (10^9^/L)		14.4 ± 10.67	16.01 ± 10.95	0.624
CRP (mg/dL)		87.35 ± 55.79	116.08 ± 69.43	0.141
Merges underlying disease	No	3	10	0.012
	Yes	20	11	
Hospitalization days in ICU		17.13 (10.00,22.00)	11.09 (3.00,14.00)	0.003

**Table 5:** △CT of miR-223-3p and miR-7110-5p between the sepsis death and survival

△CT	Survival (*n* = 23)	Death (*n* = 21)	*P* Value
miR-223-3p	1.72 ± 1.07	0.89 ± 0.24	0.056
miR-7110-5p	2.51 ± 1.02	2.01 ± 0.87	0.598

## Discussion

miRNAs have been used as biomarkers for various cancer types since the discovery of circulating miRNAs in the human peripheral sera. Several circulating miRNAs have been identified as potential biomarkers for sepsis, but their roles in infectious diseases were rarely investigated [11–17]. The association of miRNAs with the diagnosis of sepsis has been studied but remains controversial. Sepsis has various causes, such as severe wound, burn, shock, infection, and surgical operation; such causes present similarities and differences. No research has investigated the role of miRNAs in sepsis secondary to pneumonia and their relation to the diagnosis of sepsis.

In this study, the expression levels of miR-7110-5p and miR-223-3p differed among healthy controls, patients with pneumonia, and patients with sepsis secondary to pneumonia. The expression levels of these miRNAs were upregulated in the plasma of patients with sepsis secondary to pneumonia. The AUCs of the ROC of miR-7110-5p and miR-223-3p for the prediction of sepsis secondary to pneumonia were 0.883 and 0.964, respectively. This finding indicated the potential use of these miRNAs as biomarkers for sepsis.

In 2009, Vasilescu [11] first reported that miR-150 in the plasma can be used as a serum biomarker to diagnose patients with sepsis. The expression profiles of miR-150, miR-182, miR-342-5p, and miR-486 were used to distinguish patients with sepsis from healthy controls through genome-wide miRNA profiling by using microarray analysis of the peripheral blood leukocytes of eight healthy individuals and eight patients with sepsis. This result was confirmed through qRT-PCR analysis. Xie [13] screened sepsis biomarkers in the entire genome and evaluated the relationship of miRNAs to the prognosis of sepsis. The expression levels of miR-223, miR-15a, miR-122, miR-193, and miR483-5p significantly differed between the surviving and deceased group of patients with sepsis. The combination of six miRNAs with acute physiology and chronic health score (Acute Physiology Chronic Health Evaluation II) predicts sepsis and provides a sensitivity of 96.9% [16].

In this study, gene chip analysis revealed that six miRNAs, namely, miR-4800-5p, miR-6510-5p, miR-6740-5p, miR-7110-5p, miR-765, and miR-940, satisfied the screening criteria of *p* < 0.01, fold change ≥2 or < 0.5, and copy number > 50. Limited data have been presented regarding the relationship of these six miRNAs to sepsis because of the following factors: 1) sepsis displays various causes, and sepsis secondary to pneumonia has yet to be investigated in terms of the association of miRNAs with the diagnosis of sepsis; 2) unlike in previous studies, we employed Sepsis 3.0 to establish the diagnosis of sepsis; 3) the number of samples in the gene chip analysis was limited. Four miRNAs associated with sepsis in previous reports were verified through qRT-PCR analysis to compensate for the limitations of our study.

In this study, miR-223-3p was highly expressed in the circulating blood of patients with sepsis secondary to pneumonia. Wang et al. [12] compared the levels of miR-223 among 50 patients with sepsis, patients with systemic inflammatory response syndrome (SIRS), and healthy controls. The miR-223 expression level increased in the blood of patients with sepsis and SIRS caused by infection but not in the blood of patients with non-infectious SIRS. Hence, miR-223 can be used as a biomarker to distinguish infectious SIRS from non-infectious SIRS. A cohort study involving children with sepsis and healthy ones revealed that the expression levels of miR-223 and miR-146a substantially increased; the increase in miR-223 level was positively associated with a high level of tumor necrosis factor-α, disease severity, and poor prognosis [18]. However, these conclusions are inconsistent with other published reports. Clinical studies on non-infectious critically ill patients and patients with sepsis indicated that miR-223 levels with no significantly difference [19] . Our previous studies on the efficacy of miR-223-3p in predicting sepsis secondary to pneumonia revealed that the AUC of the ROC curve was 0.964, thereby suggesting that the miR-223-3p expression level can be an accurate predictor for the diagnosis of sepsis. In the present study, the expression of miR-7110-5p, which has been rarely examined, was upregulated in the circulating blood of patients with sepsis secondary to pneumonia. However, the signal of miR-7110-5p was altered not only because of the presence of pneumonia or sepsis but also possibly because of the conditions induced by pneumonia or sepsis. Fourteen miRNAs differed in terms of their expression levels in cancer stem cells, CD133(+) A549 cells, and CD133(−) cells. Among these miRNAs, five were upregulated (hsa-miR-23b-3p, −23a-3p, −15b-5p, − 24-3p, and − 4734), whereas nine were downregulated (hsa-miR-1246, −30b-5p, − 5096, − 6510-5p, has-miR-7110-5p, − 7641, − 3197, − 7108-5p, and − 6791-5p). Although miR-7110-5p was downregulated [20], its relationship to sepsis has not been studied.

This research presented several limitations. In the RT-PCR validation of miRNAs, we enrolled 52 patients with pneumonia, 44 patients with pneumonia who satisfied the Sepsis 3.0 diagnostic criteria, and 21 healthy controls. The total number of cases enrolled in the study was limited. Hence, the value of miR-7110-5p and miR-223-3p in predicting early sepsis secondary to pneumonia should be further evaluated using large samples.

## Conclusion

Agilent miRNA chip technology was applied to identify differentially expressed miRNAs in the plasma of patients with pneumonia and sepsis secondary to pneumonia (Sepsis 3.0). qRT-PCR results revealed that miR-7110-5p and miR-223-3p were upregulated in the plasma of patients with sepsis secondary to pneumonia. This study identified miR-223-3p as a promising biomarker for sepsis due to pneumonia. These results could serve as basis for future study design with an adequate sample size to verify the clinical usefulness of miRNAs as respiratory sepsis biomarkers.

## Abbreviations

AUC: Area under the curve
CAP: Community-acquired pneumonia
CV: Coefficient of Variance
HAP: Hospital-Acquired Pneumonia
miRNA: MicroRNA
MODS: Multiple Organ Dysfunction Syndrome
qRT-PCR: Quantitative Reverse-Transcription Polymerase Chain Reaction
ROC: Receiver operating characteristic curve
SIRS: Systemic Inflammatory Response Syndrome
SOFA: Sequential Organ Failure Assessment
